# Oxygen gradients in tumor tissues implications for breast cancer metastasis – a narrative review

**DOI:** 10.1097/MS9.0000000000003121

**Published:** 2025-05-12

**Authors:** Emmanuel Ifeanyi Obeagu

**Affiliations:** Department of Biomedical and Laboratory Science, Africa University, Mutare, Zimbabwe

**Keywords:** breast cancer, hypoxia-inducible factors, metastasis, oxygen gradients, tumor microenvironment

## Abstract

Oxygen gradients within tumor tissues play a pivotal role in breast cancer metastasis, influencing critical biological processes that contribute to disease progression. Tumors often develop regions of hypoxia due to rapid growth and insufficient blood supply, which drives adaptation mechanisms that promote metastatic behavior. These low-oxygen areas trigger the activation of hypoxia-inducible factors (HIFs), which regulate genes involved in angiogenesis, epithelial-to-mesenchymal transition (EMT), and immune evasion. Such processes increase the capacity of cancer cells to migrate, invade distant organs, and survive under harsh conditions, contributing significantly to metastasis in breast cancer. The interaction between oxygen gradients and the tumor microenvironment (TME) is complex, with hypoxia inducing significant alterations in immune responses. Hypoxic regions of tumors often foster an immunosuppressive microenvironment by recruiting regulatory immune cells that inhibit the function of cytotoxic T cells and natural killer cells. This immune evasion not only allows the tumor to grow but also facilitates the spread of cancer cells to secondary sites. Furthermore, hypoxia-induced angiogenesis provides the necessary vasculature for metastatic cells to enter the bloodstream and seed distant organs, further enhancing the metastatic potential of breast cancer.

## Introduction

Breast cancer remains one of the most prevalent and deadly cancers worldwide, with metastasis being the primary cause of mortality in affected patients. Metastasis is a complex, multistep process in which cancer cells disseminate from the primary tumor to distant organs, evading immune surveillance and establishing secondary growths. While much focus has been placed on the genetic and molecular pathways driving metastasis, the role of the tumor microenvironment (TME) in this process has gained increasing attention. Among the many factors within the TME, oxygen gradients are one of the most influential and poorly understood components that significantly contribute to tumor progression, particularly in metastatic breast cancer^[1]^. Tumors, due to their rapid growth, often outstrip the ability of surrounding blood vessels to deliver sufficient oxygen, resulting in the development of hypoxic regions. These areas of low oxygen create a selective pressure that influences the behavior of cancer cells, altering their metabolism, gene expression, and interactions with the surrounding stroma. Hypoxia is a hallmark of solid tumors, and its presence in breast cancer is associated with more aggressive disease, poor prognosis, and increased likelihood of metastasis. As such, understanding how oxygen gradients influence metastatic progression is crucial for developing therapeutic strategies that target the root causes of breast cancer spread^[2]^. The oxygen gradient within a tumor is not uniform but rather exists in a complex, dynamic state, where some areas experience complete or near-complete oxygen deprivation (hypoxia), while others may be mildly hypoxic or normoxic. This variability plays a key role in shaping the biology of the tumor. At the molecular level, low oxygen levels trigger the activation of hypoxia-inducible factors (HIFs), which are transcription factors that coordinate a wide array of adaptive responses to hypoxia. These factors regulate the expression of genes involved in various processes that are critical to cancer progression, including angiogenesis, epithelial-to-mesenchymal transition (EMT), immune evasion, and metabolism^[3]^.

**Highlights**

- Hypoxia induces critical molecular pathways, including HIF stabilization, that promote breast cancer metastasis via angiogenesis, EMT, and invasion.
- Targeting hypoxia-driven mechanisms offers a promising therapeutic approach for metastatic breast cancer.
- HIFs play a crucial role in the adaptive response of breast cancer cells to low-oxygen conditions, enhancing their metastatic potential.
- The tumor microenvironment, shaped by hypoxia, influences immune responses and tumor progression, offering opportunities for immune-modulating therapies.
- Combination therapies targeting hypoxia and angiogenesis could provide synergistic benefits in inhibiting breast cancer metastasis.

Angiogenesis, the formation of new blood vessels, is one of the most significant responses to hypoxia. Under low-oxygen conditions, HIFs induce the expression of vascular endothelial growth factor (VEGF), a key regulator of blood vessel formation. This vascularization allows the tumor to acquire the necessary nutrients and oxygen required for continued growth, but it also facilitates the entry of cancer cells into the bloodstream, promoting metastasis. However, the newly formed blood vessels are often aberrant, poorly structured, and leaky, creating a permissive environment for cancer cells to escape into circulation and travel to distant organs^[4]^. In addition to angiogenesis, hypoxia influences another key process in metastasis: EMT. EMT is a biological process in which epithelial cells lose their polarity and adhesion properties, gaining migratory and invasive characteristics typical of mesenchymal cells. Hypoxia promotes EMT through the stabilization of HIFs, which in turn activate signaling pathways that induce the expression of EMT-related genes. This transition facilitates the detachment of cancer cells from the primary tumor, allowing them to invade surrounding tissues and migrate to distant sites^[5]^. The influence of hypoxia on immune evasion is another crucial factor in breast cancer metastasis. Hypoxic conditions in the TME alter the immune landscape by promoting the recruitment of immunosuppressive cells, such as regulatory T cells (Tregs) and tumor-associated macrophages (TAMs). These cells release factors that inhibit the activity of cytotoxic T cells and natural killer cells, which are crucial for detecting and eliminating metastatic cells. In this way, hypoxia not only helps tumor cells survive but also contributes to the evasion of immune surveillance, further enhancing the metastatic potential of the tumor^[6]^.

The metastatic cascade is further complicated by the fact that hypoxia also influences the extracellular matrix (ECM), the structural network that surrounds and supports cancer cells. Hypoxia induces the secretion of matrix metalloproteinases (MMPs), enzymes that degrade the ECM, facilitating the invasion of cancer cells into surrounding tissues and enhancing their ability to metastasize. These ECM modifications are not only essential for local invasion but also facilitate the spread of tumor cells through the bloodstream and to distant organs^[7]^. While the role of oxygen gradients in metastasis is increasingly recognized, therapeutic strategies targeting these gradients remain in their infancy. Current approaches aim to either normalize the tumor vasculature to improve oxygen delivery or to exploit the hypoxic environment to selectively target cancer cells with hypoxia-activated prodrugs (HAPs). Additionally, HIF inhibitors are being explored as a means of blocking the adaptive responses to low oxygen, including angiogenesis and EMT. Despite the promise of these strategies, challenges remain in selectively targeting hypoxic regions of the tumor without harming normal tissue. The complexity of hypoxia in the TME, combined with the heterogeneity of oxygen distribution, presents significant obstacles to the effective implementation of these therapies^[8,9]^.

## Aim

The aim of this review is to explore the role of oxygen gradients, particularly hypoxia, in the progression and metastasis of breast cancer.

## Justification of the review

The justification for this review lies in the growing recognition of tumor hypoxia as a critical factor in the pathogenesis and metastasis of breast cancer. Despite advancements in breast cancer therapies, metastasis remains the leading cause of mortality in patients, underscoring the need for novel strategies that target the underlying mechanisms driving cancer spread. Hypoxia within the TME is a central player in this process, influencing not only tumor progression but also resistance to conventional therapies, such as chemotherapy and radiation. Currently, there is a gap in comprehensive reviews that specifically address the implications of oxygen gradients on breast cancer metastasis. This review aims to fill that gap by synthesizing current knowledge on how hypoxia drives metastasis and resistance to treatment, highlighting the potential for targeting these pathways to improve therapeutic outcomes. By providing insights into novel therapeutic approaches, such as HIF inhibitors, anti-angiogenic therapies, and strategies aimed at reprogramming tumor metabolism, this review could aid in the development of more effective treatment regimens for breast cancer patients, particularly those with advanced or metastatic disease. Moreover, it will offer a clearer understanding of the challenges and opportunities in targeting the hypoxic TME, making it a valuable resource for researchers and clinicians in the field of oncology.^[4–6]^

## Review methods

The process of reviewing the existing literature on “Oxygen Gradients and Tumor Biology: Implications for Breast Cancer Metastasis” was conducted following systematic and structured methodologies to ensure thoroughness, reproducibility, and transparency. The following review methods outline the steps taken in gathering, analyzing, and synthesizing the relevant studies and findings.

## Literature search strategy

A comprehensive literature search was conducted using electronic databases, including PubMed, Scopus, and Google Scholar. The search was performed using a combination of relevant keywords, such as “hypoxia,” “oxygen gradients,” “tumor microenvironment,” “breast cancer,” “metastasis,” and “hypoxia-inducible factors (HIFs).” The search was restricted to articles published in peer-reviewed journals within the last 10 years to ensure the inclusion of the most recent findings. Both primary research articles and review papers were considered to provide a comprehensive overview of the current state of knowledge.

Inclusion and exclusion criteria:

Studies were selected based on the following inclusion criteria:

- Original research articles, clinical trials, and review papers published in English.
- Studies focused on the role of hypoxia, oxygen gradients, and HIFs in breast cancer metastasis.
- Papers exploring therapeutic strategies targeting hypoxia in breast cancer.

Exclusion criteria includes:

- Non-peer-reviewed articles, abstracts, and editorials.
- Studies not specifically related to breast cancer or metastatic processes.
- Articles with limited relevance to the mechanisms and therapeutic implications of hypoxia in cancer.

## Oxygen gradients and tumor biology

In solid tumors, oxygen gradients are established due to the imbalance between oxygen consumption by rapidly proliferating cancer cells and the limited supply of oxygen through the existing blood vessels. These gradients create areas of both normoxia – normal oxygen levels – and hypoxia – low oxygen levels – within the tumor. Tumors typically outgrow their blood supply, leading to hypoxic regions in the interior. The outer parts of the tumor are generally well-oxygenated due to proximity to blood vessels, while the central areas experience a lack of oxygen due to limited vascularization. The resulting oxygen tension gradient plays a pivotal role in influencing tumor biology, including cancer cell survival, proliferation, invasiveness, and response to treatment^[1]^. Hypoxia within the TME is associated with various changes in cellular metabolism and behavior. In normoxic conditions, oxygen is used in mitochondrial oxidative phosphorylation to produce energy. However, in hypoxic areas, tumor cells often shift to anaerobic glycolysis, even in the presence of oxygen, a phenomenon known as the Warburg effect. This metabolic reprogramming allows cancer cells to adapt to low oxygen levels by producing ATP through glycolysis, albeit less efficiently than oxidative phosphorylation. This switch not only supports tumor growth in hypoxic regions but also contributes to the aggressive behavior of tumor cells^[2]^. The presence of oxygen gradients also influences the activation of key signaling pathways that enable tumors to adapt to the harsh microenvironment. One of the primary mediators of the hypoxic response is the HIF family of transcription factors, including HIF-1α and HIF-2α. Under low-oxygen conditions, HIFs stabilize and activate the transcription of genes involved in critical processes such as angiogenesis – formation of new blood vessels, glycolysis, and cell survival. By promoting angiogenesis, HIFs help tumors secure an adequate oxygen supply, allowing them to continue growing. Moreover, HIF activation is linked to other tumor-promoting processes such as the EMT, invasion, and metastasis, which further contribute to the aggressive nature of hypoxic tumors. Therefore, oxygen gradients not only shape the tumor’s metabolic landscape but also regulate key molecular mechanisms that enable cancer cells to survive, proliferate, and spread^[3,4]^.

## Hypoxia and breast cancer metastasis

Hypoxia, a hallmark of solid tumors including breast cancer, plays a pivotal role in the progression and metastasis of the disease. As tumors grow, the rapid proliferation of cancer cells often outpaces the development of new blood vessels, resulting in areas of oxygen deprivation within the tumor. These hypoxic conditions drive adaptive changes in tumor cells that promote survival and facilitate their ability to spread to distant organs. The process of metastasis involves cancer cells detaching from the primary tumor, invading surrounding tissues, entering the bloodstream, and eventually establishing secondary tumors in distant organs. Hypoxia within the primary tumor significantly enhances several stages of this complex process, contributing to the aggressive nature of metastatic breast cancer^[5,6]^. One of the key mechanisms by which hypoxia drives metastasis is through the stabilization and activation of HIFs, which are critical transcription factors that regulate the tumor’s response to low oxygen conditions. Under normoxic conditions, HIFs are rapidly degraded; however, in hypoxic regions, they accumulate and activate the expression of genes involved in angiogenesis, cell survival, and invasion. In breast cancer, HIF-1α and HIF-2α are particularly important in driving metastatic progression. These factors promote the expression of genes, like VEGF, which enhances blood vessel formation, and MMPs, which degrade the extracellular matrix and facilitate the migration of tumor cells into surrounding tissues. HIFs also regulate the EMT, a process that enables epithelial tumor cells to acquire mesenchymal characteristics, which enhance their motility and invasive capabilities, key steps in metastasis^[7,8]^. Hypoxia also influences breast cancer metastasis by altering the TME. Hypoxic areas of tumors are often associated with increased levels of inflammatory mediators and immune cells, such as TAMs, which can support tumor progression and metastasis. These immune cells release cytokines and growth factors that further promote the migration of cancer cells and their ability to colonize distant tissues. Additionally, hypoxia leads to metabolic reprogramming in breast cancer cells, shifting from oxidative phosphorylation to anaerobic glycolysis – the Warburg effect, which not only supports survival in low-oxygen conditions but also produces metabolic byproducts that can alter the immune landscape and enhance tumor cell invasiveness. Thus, hypoxia not only directly affects the behavior of breast cancer cells but also modifies the surrounding environment to favor metastatic spread^[9,10]^.

## The molecular mechanisms underlying hypoxia-induced metastasis

The relationship between hypoxia and metastasis is a critical aspect of tumor biology, with oxygen deprivation playing a central role in enabling cancer cells to invade, survive, and spread to distant organs. Hypoxia, a hallmark of solid tumors, results from an imbalance between oxygen supply and the rapid growth of tumor tissue. This condition induces a cascade of molecular and cellular responses that directly contribute to the metastatic process. These mechanisms include the activation of HIFs, the alteration of cellular metabolism, the induction of EMT, and the modulation of the immune microenvironment^[10]^.

### Activation of HIFs

The primary molecular regulators of the cellular response to hypoxia are the hypoxia-inducible factors, HIF-1α and HIF-2α. Under normoxic conditions, HIFs are degraded via the von Hippel–Lindau (VHL) ubiquitin–proteasome pathway. However, in hypoxic conditions, this degradation is inhibited, leading to the stabilization and accumulation of HIF-α subunits. Once stabilized, HIFs translocate to the nucleus, where they bind to hypoxia-responsive elements (HREs) in the promoter regions of target genes. These genes are involved in critical processes such as angiogenesis – via VEGF, glucose metabolism – via glycolytic enzymes, EMT (via transcription factors like Snail and Twist), and cell survival. The activation of these pathways promotes the ability of cancer cells to adapt to low oxygen, migrate, invade, and ultimately metastasize to distant sites^[11]^.

### Angiogenesis and the tumor vasculature

Hypoxia-induced angiogenesis is one of the most significant mechanisms by which tumors can overcome oxygen deprivation. The activation of HIF-1α leads to the transcription of pro-angiogenic factors, particularly VEGF. VEGF stimulates the growth of new blood vessels, allowing tumors to improve oxygen delivery. However, the newly formed blood vessels are often structurally abnormal, resulting in a leaky vasculature that facilitates the entry of cancer cells into the bloodstream. This process, known as intravasation, is critical for metastasis, as it enables the tumor to disseminate cells to distant organs. The presence of aberrant blood vessels further promotes the development of secondary tumors by providing an accessible route for circulating tumor cells (CTCs) to seed new growths in distant tissues^[12]^.

### EMT

One of the most important cellular transitions induced by hypoxia is EMT. During EMT, epithelial cancer cells lose their cell-cell adhesion properties and acquire mesenchymal traits, including increased motility, invasiveness, and resistance to apoptosis. Hypoxia induces the expression of EMT-related transcription factors, such as Snail, Twist, and Zeb1, which downregulate the expression of epithelial markers like E-cadherin and upregulate mesenchymal markers such as N-cadherin and vimentin. This shift enhances the ability of cancer cells to detach from the primary tumor mass and invade surrounding tissues, thus initiating the metastatic process. Additionally, hypoxia-induced EMT facilitates the ability of cancer cells to survive in the circulatory system, contributing to their successful extravasation and colonization at distant sites^[13]^.

### Immune evasion and TME

Hypoxia plays a critical role in modifying the immune landscape of tumors, fostering an environment that supports immune evasion and metastasis. Hypoxic regions of tumors recruit immunosuppressive cells, such as Tregs, TAMs, and myeloid-derived suppressor cells (MDSCs), which inhibit the function of anti-tumor immune cells like cytotoxic T cells and natural killer (NK) cells. The expression of immunosuppressive cytokines and immune checkpoint molecules, such as programmed death-ligand 1 (PD-L1), is also upregulated in hypoxic conditions. This immunosuppressive microenvironment allows the tumor to evade immune surveillance, providing a more permissive setting for the survival and spread of cancer cells. Furthermore, hypoxia-induced changes in immune cell function can facilitate the creation of a “metastatic niche,” where immune cells actively support the migration and colonization of cancer cells in distant organs^[14]^.

### ECM remodeling

In addition to cellular and immune changes, hypoxia also triggers remodeling of the ECM, a critical component of the TME that supports tumor growth and metastasis. Hypoxia induces the expression of MMPs, enzymes that degrade ECM components such as collagen, fibronectin, and laminin. The degradation of the ECM facilitates the invasion of cancer cells into surrounding tissues and blood vessels, thereby promoting metastasis. Moreover, the altered ECM environment can also influence the behavior of cancer-associated fibroblasts (CAFs) and other stromal cells, further enhancing the metastatic potential of tumors. These changes are often accompanied by an increase in tumor stiffness, which also contributes to the mechanical forces that drive cancer cell migration and invasion^[2]^.

### Metabolic reprogramming

Hypoxia induces significant metabolic reprogramming in cancer cells to adapt to limited oxygen availability. This metabolic shift is characterized by an increase in glycolysis, even in the presence of oxygen, a phenomenon known as the Warburg effect. In hypoxic regions, cancer cells rely more heavily on glycolysis to generate energy, even in the absence of sufficient oxygen for oxidative phosphorylation. This metabolic shift is regulated by HIFs, which upregulate the expression of glycolytic enzymes and glucose transporters. The resulting acidification of the TME can further promote tumor invasion and metastasis by enhancing the activity of proteolytic enzymes and contributing to ECM remodeling. Additionally, the metabolic reprogramming of cancer cells can provide an advantage for their survival in distant metastatic sites, where nutrient availability may be scarce^[15]^.

### Hypoxia and drug resistance

Hypoxia is also a key factor in promoting drug resistance in cancer cells. Many conventional chemotherapy agents rely on the presence of oxygen to exert their cytotoxic effects, as they typically generate reactive oxygen species (ROS) that damage cellular components. However, in hypoxic regions, cancer cells are less sensitive to ROS-induced damage due to altered metabolic processes and a reliance on anaerobic pathways. Hypoxia also leads to the upregulation of drug efflux pumps, which reduce the intracellular concentration of chemotherapeutic agents, further contributing to resistance. As a result, hypoxia often limits the efficacy of both traditional chemotherapies and targeted therapies, making it a major obstacle in the treatment of metastatic breast cancer^[16]^.

## The impact of immune evasion in hypoxic tumors

Hypoxia, a common feature of solid tumors, contributes to the malignant progression of cancer by altering both the tumor cells themselves and the surrounding TME. Among the most critical effects of hypoxia is its ability to promote immune evasion, a key mechanism by which tumors protect themselves from immune surveillance and destruction. As the tumor grows, it often outpaces the ability of blood vessels to provide sufficient oxygen, leading to regions of hypoxia. These hypoxic regions induce a cascade of molecular changes that suppress anti-tumor immunity and promote the survival of tumor cells, thereby facilitating tumor growth, metastasis, and resistance to therapy. The impact of immune evasion in hypoxic tumors is multifaceted, involving alterations to immune cell recruitment, immune checkpoint regulation, and the activation of immune-suppressive signaling pathways.

### Hypoxia-induced immune cell reprogramming

The hypoxic environment in tumors is known to alter the behavior of immune cells within the TME. TAMs, Tregs, and MDSCs are often recruited to hypoxic regions of tumors. These immune cells contribute to tumor progression by exerting immunosuppressive effects. Under hypoxic conditions, TAMs, which are usually polarized into pro-inflammatory (M1) or anti-inflammatory (M2) phenotypes, are predominantly skewed toward the M2-like phenotype. M2 macrophages secrete cytokines, such as IL-10 and TGF-β, which dampen the activity of cytotoxic immune cells like CD8+ T cells and NK cells. This M2 polarization supports tumor cell survival, angiogenesis, and tissue remodeling, thus fostering an immune-tolerant environment. Similarly, hypoxia enhances the recruitment and activation of Tregs, a subset of T cells that inhibit the function of effector T cells and maintain immune tolerance. Tregs accumulate in hypoxic tumors and produce immunosuppressive cytokines that promote immune evasion. Hypoxia also induces the expression of immunosuppressive factors, such as adenosine, which suppresses T cell activation and promotes the differentiation of Tregs. The increased presence of Tregs within the tumor microenvironment diminishes the effectiveness of anti-tumor immunity, allowing cancer cells to evade immune detection and destruction^[17]^.

### Immune checkpoint regulation in hypoxic tumors

Immune checkpoint molecules, such as programmed cell death protein 1 (PD-1) and cytotoxic T lymphocyte antigen-4 (CTLA-4), are critical modulators of immune responses in cancer. These checkpoints act to dampen the immune system’s response to self and non-self antigens, which is essential for maintaining immune tolerance but can be exploited by tumors to avoid immune detection. In hypoxic tumors, the expression of immune checkpoint ligands, particularly PD-L1, is upregulated in response to low oxygen levels. This upregulation is largely mediated by HIF transcription factors, which are stabilized under hypoxic conditions and drive the expression of genes involved in immune evasion, including PD-L1. PD-L1 binds to PD-1 on immune cells, particularly T cells, leading to T cell exhaustion and inhibition of anti-tumor responses. The upregulation of immune checkpoints such as PD-L1 in the context of tumor hypoxia can severely impair the ability of cytotoxic T cells to recognize and kill tumor cells. T cell exhaustion, characterized by decreased cytokine production, reduced proliferation, and impaired cytotoxicity, is a hallmark of chronic infection and cancer. In the tumor microenvironment, this exhaustion state is exacerbated by the hypoxic conditions, further promoting immune tolerance and enhancing tumor progression. Consequently, immune checkpoint inhibitors, such as anti-PD-1 or anti-PD-L1 antibodies, are increasingly being tested as therapeutic strategies to counteract immune evasion in hypoxic tumors and restore effective anti-tumor immunity^[18,19]^.

### Hypoxia and the tumor vasculature

In addition to modulating immune cell function, hypoxia significantly alters the tumor vasculature, which plays a pivotal role in the immune response. The hypoxic TME often leads to the formation of poorly structured and dysfunctional blood vessels. These abnormal vessels impede the proper infiltration of immune cells into the tumor, limiting the ability of immune effector cells to reach tumor cells and mount an effective response. Furthermore, the leaky nature of these blood vessels contributes to increased interstitial pressure within the tumor, which further hampers the penetration of immune cells. In hypoxic tumors, the vasculature is also characterized by the upregulation of certain adhesion molecules and pro-inflammatory cytokines, which can further contribute to immune cell trafficking and dysfunction. Hypoxia-driven signaling pathways, such as those involving VEGF, alter the permeability and structure of blood vessels, allowing cancer cells to escape into the bloodstream and metastasize. This impaired immune cell infiltration, combined with the immunosuppressive tumor microenvironment, exacerbates the immune evasion and survival of tumor cells in hypoxic conditions^[4]^.

### Impact on immunotherapy efficacy

Hypoxia-mediated immune evasion poses a significant challenge to the success of immunotherapies. Immunotherapies that rely on stimulating the immune system, such as immune checkpoint inhibitors and adoptive T cell therapies, can be less effective in hypoxic tumors due to the suppressed immune response. The presence of Tregs, MDSCs, and TAMs in hypoxic regions further hinders the ability of these therapies to promote effective anti-tumor immunity. Additionally, the poor vascularization of hypoxic tumors can limit the delivery and infiltration of immune cells and therapeutic agents, reducing the overall efficacy of immunotherapy. To overcome these challenges, researchers are exploring strategies to normalize the tumor vasculature or enhance the oxygenation of tumors. By improving blood flow and oxygen delivery, it may be possible to enhance the infiltration of immune cells and increase the efficacy of immunotherapies. Furthermore, combination therapies that target both the immune system and the tumor vasculature are being investigated to counteract the immunosuppressive effects of hypoxia and improve therapeutic outcomes^[20]^.

## Targeting oxygen gradients as a therapeutic strategy

Oxygen gradients within tumors are a hallmark of solid malignancies, especially as tumors grow and outstrip the supply of oxygen through their blood vessels. These gradients create regions of varying oxygen tension within the TME, with some areas becoming severely hypoxic. Hypoxia, in turn, drives numerous adaptive responses that promote tumor survival, metastasis, and resistance to therapies. The manipulation of these oxygen gradients – by either normalizing the vasculature to improve oxygen delivery or selectively targeting hypoxic tumor cells – has emerged as a promising therapeutic strategy. By targeting oxygen gradients, it may be possible to disrupt the tumor’s ability to adapt to low-oxygen conditions, making them more susceptible to treatment and inhibiting their malignant behavior.

### Normalizing tumor vasculature to improve oxygenation

One of the primary challenges of tumors is the abnormal structure and function of their blood vessels. These vessels are often irregular, poorly formed, and leaky, leading to areas of insufficient oxygen supply – hypoxia. This vascular dysfunction limits the effectiveness of treatments such as chemotherapy and radiation, which rely on the delivery of therapeutic agents through the bloodstream. A strategy to target oxygen gradients involves normalizing the tumor vasculature to improve oxygen delivery and enhance the efficacy of therapies. The concept of vascular normalization focuses on the restoration of proper blood vessel structure and function within the tumor. This is achieved through the use of agents that target the abnormal signaling pathways involved in angiogenesis and vessel growth, such as those mediated by VEGF. By inhibiting VEGF signaling or other angiogenic pathways, these agents can reduce the tortuosity and permeability of blood vessels, enabling more efficient oxygen and drug delivery to the tumor. Normalizing the vasculature not only improves oxygenation but also increases the sensitivity of tumors to radiation therapy and chemotherapy by enhancing the delivery of cytotoxic agents^[21]^.

### HAPs

Another approach to targeting oxygen gradients is the use of HAPs. HAPs are a class of drugs that are designed to remain inactive in normal oxygen conditions but become activated in the low-oxygen – hypoxic – regions of tumors. These prodrugs are selectively converted into their active cytotoxic forms under hypoxic conditions, thereby sparing normal, well-oxygenated tissues from toxic effects. The selective activation of these drugs in hypoxic tumor areas allows for targeted therapy, minimizing side effects and increasing the therapeutic index. HAPs exploit the fact that tumor cells in hypoxic regions have a higher metabolic rate and altered enzymatic activity that enables the activation of these prodrugs. Some of the most studied HAPs include tirapazamine, which has shown promise in combination with other therapies, such as chemotherapy and radiation, to effectively kill hypoxic tumor cells. By targeting the oxygen gradients within tumors, HAPs provide a direct means to eradicate cells that would otherwise be resistant to standard treatments. These drugs offer a unique strategy for overcoming the challenges posed by hypoxic regions, which are often more difficult to treat due to their reduced blood supply^[22]^.

### Targeting HIF pathways

HIFs are key transcriptional regulators that mediate the cellular response to hypoxia. In response to low oxygen levels, HIFs activate a wide array of genes involved in cell survival, angiogenesis, metabolism, and immune modulation. The most studied of these factors, HIF-1, is often overexpressed in tumors, particularly in regions that experience chronic hypoxia. The activation of HIF-1 leads to the transcription of genes such as *VEGF, GLUT1* (glucose transporter 1), and *BNIP3* (Bcl-2/adenovirus E1B 19 kDa-interacting protein 3), which promote angiogenesis, glucose metabolism, and cell survival under hypoxic conditions. Targeting the HIF pathways is an attractive therapeutic strategy for interrupting the adaptive responses of tumor cells to hypoxia. Several HIF inhibitors have been developed, including small molecules and antibodies that block HIF-1α stabilization or the binding of HIF-1 to its DNA target sites. By inhibiting HIF-1 activity, it is possible to suppress the tumor’s ability to adapt to hypoxic conditions, hinder angiogenesis, and increase the sensitivity of tumor cells to therapies. However, challenges remain in selectively targeting HIFs without affecting normal tissue responses to hypoxia, as HIFs are also involved in important physiological processes in healthy tissues^[23,24]^.

### Combination therapies to overcome hypoxia-induced resistance

Tumor hypoxia is a significant driver of resistance to therapies, such as radiation, chemotherapy, and immunotherapy. Radiation therapy, for example, is less effective in hypoxic tumor regions due to the reduced ability of oxygen to enhance the formation of DNA-damaging free radicals. One approach to overcoming this resistance is through combination therapies that target both the tumor oxygen gradients and the tumor cells themselves. One such strategy is the use of hypoxia-targeting agents in combination with conventional therapies. For instance, HAPs can be used in combination with chemotherapy to specifically target hypoxic tumor cells that are resistant to conventional treatments. Similarly, anti-angiogenic therapies, which normalize tumor vasculature, can be combined with radiation or chemotherapy to improve oxygenation and increase treatment efficacy. Another promising combination approach involves the use of immune checkpoint inhibitors alongside vascular normalization or HAPs. By enhancing oxygen supply and targeting hypoxic cells, these combination therapies may help to overcome the immune evasion associated with hypoxia and improve the overall anti-tumor immune response^[25]^.

## Future prospectives

The therapeutic targeting of oxygen gradients within tumors holds substantial promise for improving cancer treatment, but numerous challenges must be addressed before these strategies can be fully integrated into clinical practice. Looking ahead, several key advancements could shape the future of oxygen-gradient targeting in cancer therapy, particularly in breast cancer metastasis.

### Precision medicine and biomarkers

As tumors exhibit significant heterogeneity in their oxygen gradients, one critical area for future development is the identification and validation of biomarkers that can accurately assess the degree of hypoxia within individual tumors. Advances in imaging technologies, such as positron emission tomography (PET) and magnetic resonance imaging (MRI), as well as the development of novel hypoxia-sensitive biomarkers, will enable the precise identification of hypoxic regions. These tools could help clinicians tailor oxygen-targeting therapies to specific tumor subtypes and patient profiles, maximizing therapeutic efficacy and minimizing unnecessary side effects. In the context of precision medicine, combining oxygen-gradient targeting with genomic and proteomic profiling could provide a more comprehensive understanding of tumor behavior, enabling the design of highly individualized treatment regimens^[26,27]^.

### Overcoming resistance mechanisms

Tumor cells can adapt and evolve in response to therapies, including those targeting oxygen gradients. Resistance mechanisms to hypoxia-targeting therapies, such as the overexpression of alternative angiogenesis pathways or adaptive shifts in metabolism, are likely to arise. The future of oxygen-gradient targeting will involve the development of strategies that not only target hypoxic areas but also prevent or overcome these resistance mechanisms. For instance, combining hypoxia-targeting agents with drugs that inhibit specific resistance pathways, such as inhibitors of alternative angiogenesis factors or metabolic modulators, could enhance the long-term efficacy of treatment. A multi-faceted approach addressing both tumor microenvironmental changes and cellular adaptations will be crucial in overcoming the challenges posed by resistance^[28,29]^.

### Immunotherapy synergy

Hypoxic tumors are often immune-resistant, as they can suppress immune cell infiltration and promote immune evasion through mechanisms like the upregulation of immune checkpoints and the recruitment of immunosuppressive cells. However, recent breakthroughs in immunotherapy have shown the potential to overcome these immune barriers. Future research should focus on combining oxygen-gradient targeting therapies with immune checkpoint inhibitors or adoptive T cell therapies. These combination approaches could not only re-oxygenate the TME but also enhance the anti-tumor immune response, offering synergistic effects that might be particularly beneficial in aggressive cancer types, such as metastatic breast cancer^[30,31]^.

### Targeted drug delivery systems

The challenge of selectively targeting hypoxic regions within tumors without affecting normal tissues remains a significant hurdle. Advances in drug delivery systems, such as nanoparticles, liposomes, and conjugated antibodies, could provide more precise delivery of hypoxia-activated prodrugs and other therapeutic agents. By utilizing targeting ligands that specifically bind to hypoxic tumor cells or their associated blood vessels, these novel delivery systems could increase the concentration of therapeutic agents in tumor tissues while minimizing off-target effects. This approach could also improve the bioavailability and half-life of oxygen-targeting drugs, further enhancing their therapeutic potential^[32,33]^.

### Clinical translation and personalized therapy

While preclinical studies have shown great promise, the clinical translation of oxygen-gradient targeting therapies remains a work in progress. As we move forward, the emphasis will be on conducting well-designed clinical trials to assess the safety, efficacy, and long-term benefits of these approaches in human patients. Large-scale, multi-center trials will be necessary to determine the best combinations of oxygen-targeting strategies with existing therapies, such as chemotherapy, radiation, and immunotherapy. Personalized therapy, based on the specific characteristics of each patient’s tumor, including the extent of hypoxia and molecular profile, will be key to optimizing treatment outcomes. As research continues, understanding how to incorporate these therapies into standard treatment regimens for metastatic cancers, like breast cancer, will be pivotal in improving patient survival and quality of life^[34]^.

## Recommendations

### Targeting HIFs

Given the critical role of HIFs in hypoxia-driven metastasis in breast cancer, the development of HIF inhibitors or molecules that prevent HIF stabilization could provide an effective strategy to inhibit tumor growth and metastasis. Therapeutic approaches targeting HIFs should be explored in clinical trials to assess their efficacy in preventing the progression of metastatic breast cancer.

### Combination therapy with anti-angiogenic agents

As hypoxia induces angiogenesis through HIF activation, combining HIF-targeting strategies with anti-angiogenic therapies, such as bevacizumab – anti-VEGF antibody, may have synergistic effects. This combination could reduce the blood supply to tumors, making them more susceptible to other treatments like chemotherapy and radiation.

### Modulation of TME

The TME, particularly in hypoxic regions, significantly contributes to breast cancer metastasis. Targeting factors that alter the TME, such as TAMs and ECM remodeling, could provide additional therapeutic opportunities. Immunotherapies aimed at reprogramming the immune landscape of hypoxic tumors should be further investigated.

### Metabolic reprogramming as a therapeutic target

Tumor cells in hypoxic regions shift toward anaerobic glycolysis to survive. Therapeutic strategies aimed at reversing this metabolic reprogramming, such as targeting glycolytic enzymes or promoting oxidative phosphorylation, could reduce the aggressiveness of breast cancer and limit metastasis.

### Personalized approaches to target hypoxia

Given the heterogeneity of breast cancer, it is crucial to develop personalized treatment strategies that target hypoxia and its effects based on the molecular characteristics of individual tumors. Biomarkers that identify hypoxic regions in tumors, such as glucose analogs or PET imaging agents, could guide the selection of appropriate therapies tailored to the patient’s specific tumor biology.

### Preclinical models and clinical trials

Further preclinical and clinical research is needed to identify reliable biomarkers of hypoxia in breast cancer and to validate the effectiveness of hypoxia-targeting therapies. Animal models and clinical trials should focus on evaluating the efficacy of HIF inhibitors, anti-angiogenic agents, and other novel therapies aimed at reducing hypoxia-driven metastasis.

### Patient monitoring and early detection of metastasis

Since hypoxia contributes to the spread of breast cancer, regular monitoring of hypoxic conditions in patients through imaging techniques or biomarkers could help in the early detection of metastasis. Early intervention could significantly improve patient prognosis and prevent the development of advanced metastatic disease.

### Collaborative research efforts

A multidisciplinary approach involving oncologists, immunologists, biochemists, and pharmacologists is essential to advance our understanding of hypoxia’s role in breast cancer metastasis and to develop targeted therapies. Collaboration between academic institutions, pharmaceutical companies, and healthcare providers could accelerate the discovery of effective treatments.

## Conclusion

Targeting oxygen gradients in tumors presents a highly promising therapeutic strategy to address one of the most challenging features of solid malignancies – hypoxia. The unique molecular and cellular responses that tumors exhibit in response to oxygen deprivation, including metabolic reprogramming, immune evasion, and enhanced metastasis, drive therapeutic resistance and tumor progression. By focusing on the manipulation of oxygen supply within the tumor microenvironment, such as normalizing abnormal vasculature, using HAPs, and targeting HIFs, significant strides can be made in improving treatment efficacy and overcoming resistance to traditional therapies.

## Data Availability

Not applicable.
